# Advances in Understanding the Mechanism of Action of the Auxin Permease AUX1

**DOI:** 10.3390/ijms19113391

**Published:** 2018-10-30

**Authors:** Gaurav Singh, Katarzyna Retzer, Stanislav Vosolsobě, Richard Napier

**Affiliations:** 1 School of Life Sciences, University of Warwick, Coventry CV4 7AL, UK; G.Singh.7@warwick.ac.uk; 2 Institute of Experimental Botany, Czech Academy of Sciences, Rozvojova 263, 165 02 Prague 6, Czech Republic; retzer@ueb.cas.cz; 3 Department of Experimental Plant Biology, Faculty of Science, Charles University, Vinicna 5, 128 44 Prague 2, Czech Republic; stanislav.vosolsobe@natur.cuni.cz

**Keywords:** auxin transport, hormone, development, kinetics, permeability, structure

## Abstract

In over 40 years of research on the cellular uptake of auxin it is somewhat chastening that we have elaborated so little on the original kinetic descriptions of auxin uptake by plant cells made by Rubery and Sheldrake in 1974. Every aspect of that seminal work has been investigated in detail, and the uptake activity they measured is now known to be attributed to the AUX1/LAX family of permeases. Recent pharmacological studies have defined the substrate specificity of AUX1, biochemical studies have evaluated its permeability to auxin in plant cell membranes, and rigourous kinetic studies have confirmed the affinity of AUX1 for IAA and synthetic auxins. Advances in genome sequencing have provided a rich resource for informatic analysis of the ancestry of AUX1 and the LAX proteins and, along with models of topology, suggest mechanistic links to families of eukaryotic proton co-transporters for which crystal structures have been presented. The insights gained from all the accumulated research reflect the brilliance of Rubery and Sheldrake’s early work, but recent biochemical analyses are starting to advance further our understanding of this vitally important family of auxin transport proteins.

## 1. Introduction

Auxin regulates manifold plant developmental and stress adaption processes [1], and so the proper and balanced arrangement of auxin synthesis, storage, degradation and transport is vital. There exist both specific and non-specific mechanisms of transit across the plasma membrane (PM), and in particular auxin is actively transported by influx and efflux carriers to control spatial and temporal delivery and ensure optimal plant growth [1,2]. The most studied influx carriers in *Arabidopsis thaliana* are the members of the AUXIN1/ LIKE-AUX1 (AUX1/LAX) family, which includes the AUX1 and LAX1-3 proteins [3], although at least two ATP-Binding Cassette sub-Family B (ABCB) carriers and the nitrate transporter NRT1.1 have also been shown to contribute to auxin uptake [4,5,6,7]. Other ABCBs and PIN-formed (PIN) proteins coordinate efflux of auxin. However, in this review we will focus on the AUX1 family and in particular on the biochemistry of AUX1/LAX proteins.

The cell biology and physiology associated with AUX1/LAX proteins have been the subject of thorough reviews [3] and so only a brief summary of the physiology of AUX1-related responses is given here as a prelude to a more thorough focus on the biochemistry of AUX1. AUX1 is crucial for the asymmetric distribution of auxin in the gravistimulated root tip [8], and in combination AUX1 and LAX3, regulate auxin levels to initiate lateral root (LR) development and apical hook formation [3,9,10,11]. Furthermore, AUX1, LAX1 and LAX2 are required for proper leaf development [3,12] and AUX1 is required for root hair elongation in response to low phosphate [13]. In general, AUX1/LAX proteins mediate the flow of auxin through the plant body and assist in generating auxin maxima and minima. Moreover, recent research on very rapid responses to auxin suggest that AUX1 activity may underpin a wider range of physiological responses [14,15]. 

## 2. Early Discoveries and the Chemiosmotic Theory for Auxin Accumulation

**Figure 1:**
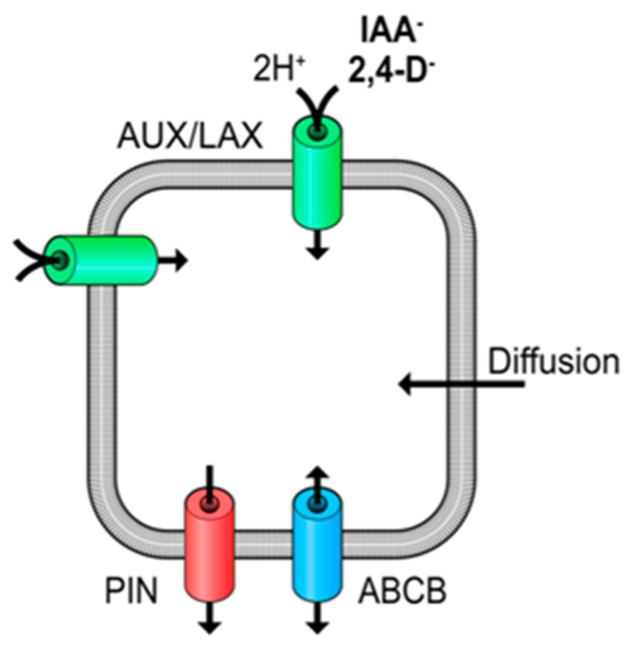
Schematic summary of the pathways of auxin transport in a plant cell.

Natural and synthetic auxins are weak acids (e.g., indole-3-acetic acid, IAA p*Ka* = 4.7) and, as a consequence, a significant proportion will be protonated and uncharged at regular apoplast pH values of between 4.5 and 5.5 [16]. This may allow the molecule to diffuse across the PM, and this diffusive flux is widely recognized, although cellular uptake via auxin transport proteins is also necessary and vitally important (Figure 1) [17,18]. The cytoplasm has a pH close to neutral, and at this pH IAA will be deprotonated and become anionic, trapped in the cell by the well-known anion trap and dependent on transport proteins for exit [17]. The seminal work of Rubery and Sheldrake [17] uncovered most features of the mechanisms of auxin transport that we understand today. They used plant cell suspension cultures to follow the accumulation kinetics of radiolabelled auxin and showed that uptake was pH-dependent. This led them to propose that there is probably IAA-proton co-transport. Further, they showed that uptake was saturable and, hence, carrier mediated. Moreover they estimated the half saturation concentrations for IAA and 2,4-D to be low micromolar, and they calculated that the anion trap would concentrate IAA^−^ in the cytoplasm above the concentrations of IAA in both apoplast and vacuole. In this review we will discuss what details have been added to these early discoveries.

## 3. Auxin Uptake Carriers Are Proton Co-Transporters

The auxin uptake carriers use both membrane potential and protons to drive uptake. Exploration of the chemiosmotic theory using accumulation of radiolabelled auxin in suspension cultured crown gall cells confirmed the dependence of uptake on protons [19]. At around the same time, experiments on the mechanism of auxin perception were using membrane preparations from *Cucurbita pepo* hypocotyls, and one of the auxin-binding fractions (known as auxin-binding site III) [20] was soon shown to be associated with uptake into sealed membrane vesicles. This accumulation of radiolabelled IAA inside these vesicles was shown to be driven by pH gradients and collapsed by protonophores [21]. Consequently, site III “binding” was shown to be auxin uptake activity, not binding, and thereafter, the use of microsomal vesicles simplified transport experiments by avoiding the competing contributions of subcellular compartments inherent with intact cells. Many key biochemical features of the auxin efflux system were characterized in these early works, not least because chemical inhibition of efflux using e.g., naphthylphthalamic acid (NPA) increased net accumulation and facilitated interpretation of the data. 

The *Cucurbita* vesicle system leant itself to a detailed examination of the chemiosmotic drivers. By incorporating electron paramagnetic spin probes into the vesicles both the pH gradient and vesicle volume could be measured and, hence, the stoichiometry of proton and IAA transport [22]. This work confirmed that two protons were co-transported with each IAA^−^ anion and that the pH gradient supporting two-proton co-transport is necessary and sufficient, even though both earlier and later work showed that hyperpolarization of the plasma membrane can further stimulate auxin accumulation [21,23]. This hyperpolarisation indicated an additional, voltage-dependent component controlling uptake activity [21,23]. Later, extracellular calcium was shown to give a strong stimulation of IAA accumulation in moss protoplasts [24]. Given that rising intracellular calcium appears to be a rapid response to auxin in root hairs [14], this element of accumulation control merits confirmation. It is also of interest to note the feedback of auxin uptake on apoplastic pH and vice versa [25,26]. Uptake may raise apoplastic pH via symport of protons with IAA through AUX1 (Figure 1) and, in roots, auxin uptake also regulates PM H^+^ATPase activity at low concentrations [15], pumping the protons back outside.

## 4. AUX1 and LAX Proteins Identified as Auxin Transporters

**Figure 2:**
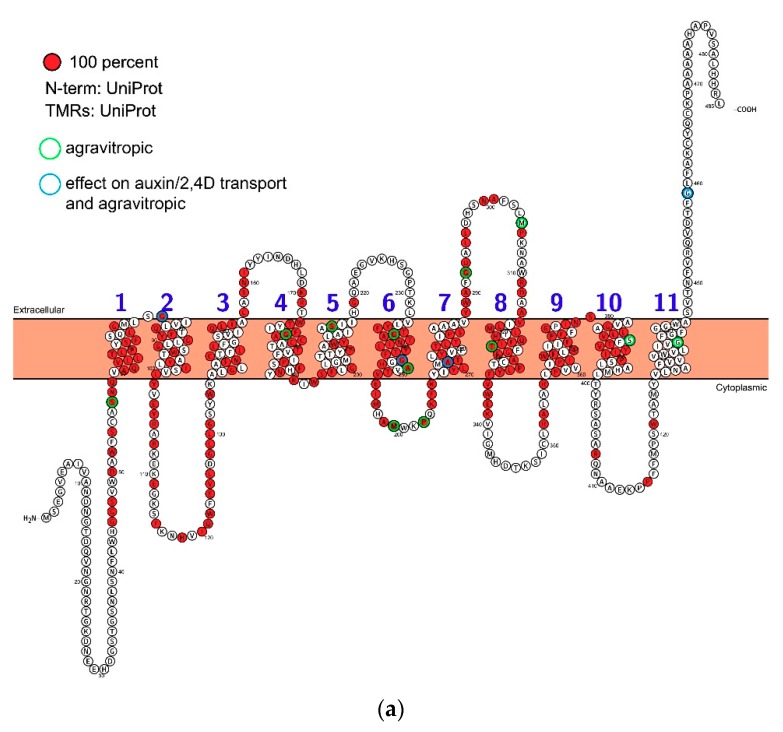

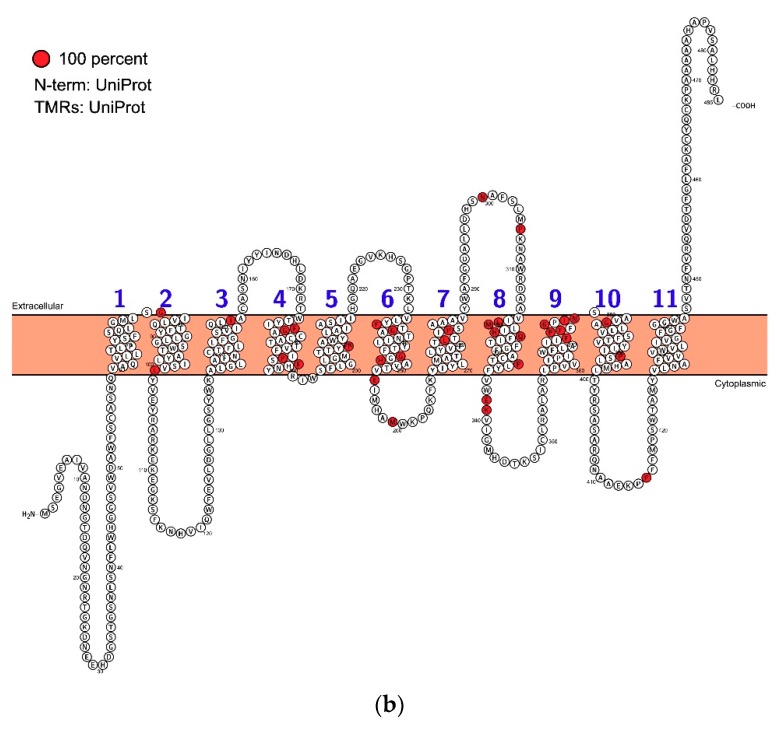
The topology and residue conservation of AUX1. Based on alignment analysis using ClustalW a topological representation of the protein was created using Protter [34]. (**a**) Land plant sequences. Residues coloured red are 100% conserved. The sites of mutations are indicated by circles around relevant residues. (**b**) As for a, but including the sequence data for *Chlorella* in the analysis.

All the fundamental characteristics of the IAA uptake system described above were discovered without knowledge of the carrier proteins. It was not until 1996 when AUX1 was identified as a candidate uptake transporter (Figure 2) [27], even though the first At*aux1* mutants were described in 1980 [28] in a screen for auxin (2,4-dichlorophenoxyacetic acid, 2,4-D) resistance. They were found to have agravitropic root growth, but otherwise the phenotype was not remarkable. Further *aux1* mutant lines were identified [29], summarized and reviewed in [3,30]. Phenotype analyses led onto the discoveries that *aux1* mutants were defective in basipetal auxin transport [8] and the revelation that AUX1 is an auxin uptake carrier. The failure of appropriate auxin distribution in *aux1* lines explained the agravitropic phenotype. No other member of the AUX1/LAX family plays a role in root gravitropism [31] and *lax* phenotypes are far subtler. Among the most striking are *lax2*, with impaired development of veins in cotyledons [31] and *lax3* showing deficiencies in apical hook formation [10]. It took some years before measurements of carrier-mediated IAA accumulation *in planta* showed that AUX1 is a high-affinity IAA importer. For example, the rate of AUX1-mediated uptake was shown to be 15-times greater than the rate of IAA movement by diffusion when AUX1 is expressed in root epidermal cells [32,33].

With the identification of the gene for AUX1, heterologous expression in several eukaryotic expression systems allowed a reductionist evaluation of its properties. For example, expression of AtAUX1 in *Xenopus* oocytes followed by radiotracer accumulation assays gave us kinetic parameters with a Michaelis constant, K_M_ for IAA of 0.84 µM [35]; IAA uptake was shown to be pH-dependent with a pH optimum around pH 6; IAA uptake was abolished if knock-out mutant versions of *AUX1* were expressed; IAA uptake was competed by 2,4-D, and known inhibitors of auxin uptake mimicked this activity in oocyte assays [35]. In similar work using baculovirus-mediated expression in insect cells, the equilibrium dissociation constant, K_D_, for IAA was estimated as 2.6 µM [36]. No expression studies using LAX proteins have been reported and it will be interesting to compare affinities and selectivities across the family.

## 5. Selectivity of the Auxin Uptake System

Heterologous expression of AUX1 also gave the opportunity to evaluate its selectivity. A few synthetic auxins were tested as substrates, but generally at single, high concentrations and the results reinforced knowledge that e.g., the naphthoxyacetic acids (NOAs) competed with IAA uptake [37], and that 2,4-D was a very good substrate [31]. However, much more information on selectivity has been collected from studies using radiolabelled IAA accumulation into suspension cultured tobacco cells [38,39,40,41]. Early pharmaceutical studies of carrier-mediated auxin uptake had been hampered by the absence of suitable inhibitors. Delbarre’s group screened compounds based on aryl and aryloxyalkylcarboxylic auxin scaffolds and produced a 2-dimensional molecular grid which described compounds likely to be substrates for uptake carriers, and those that would be excluded [39]. Where early work used 2-naphthalene acetic acid (2-NAA) to inhibit AUX1 activity [42], later both 1-naphthoxy acetic acid (1-NOA) and 3-chloro-4-hydroxyphenylacetic acid (CHPAA) became widely used [38]. Both 1-NOA and CHPAA have been investigated *in planta* and shown e.g., to phenocopy the agravitropism seen with *aux1* mutants [18]. More recent discoveries have been the 5-alkoxy derivatives of IAA and 7-alkoxy derivatives of 1-NAA as potent inhibitors of auxin transport, although these are non-selective inhibitors of all classes of auxin transporters [43].

**Figure 3:**
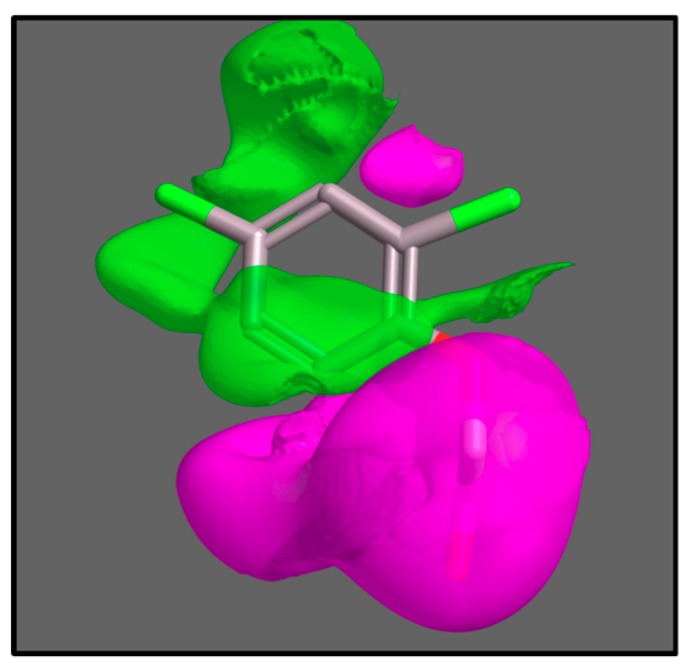
The AUX1 pharmacophore. The pharmacophore is a three-dimensional map of the characteristics of all known substrates of AUX1 [44]. The activity cliff map for hydrophobicity is shown with the structure of 2,4-D inside as a reference compound. The volumes coloured green indicate spaces where additional hydrophobicity increases activity (compounds filling these spaces with hydrophobic groups are better substrates for AUX1); the volumes coloured magenta indicate spaces where hydrophobicity decreases activity.

Delbarre’s substrate grid persisted for many years until recent computational chemistry combined with mathematical modelling of uptake kinetics were used to give three-dimensional molecular field maps of AUX1 selectivity, along with accurate estimations of relative affinities of the AUX1 protein [44]. Molecular field maps present the dimensions and biophysical properties of the average perfect AUX1 substrate, and can be used in computational screens for compounds which will be carried by AUX1, and those which will not (Figure 3).

## 6. Uptake Selectivity and Auxin Herbicide Activity

The pharmacophore study [44] included all of the compound scaffolds used for synthetic auxins, several of which had not been assessed for uptake previously. Somewhat surprisingly, several classes of synthetic auxin used widely as herbicides were found not to be substrates, indicating that AUX1 is not necessary for cells to accumulate herbicidal concentrations of auxins. As mentioned above, auxins have acidic p*Ka* values meaning that in the apoplast a significant percentage is protonated (with no net charge). Protonated auxins will be dominated by hydrophobic aromatic ring systems, making it possible for them to permeate or diffuse into and across cell membranes (Figure 1). Indeed, every accumulation assay has observed a non-saturable component and this is, invariably, referred to as diffusion and explained by this lipophilicity at lower pH values [17,40,45]. Such a biophysical process also readily explains how auxin herbicides can enter the cell and accumulate. However, there are elegant arguments which counter the association of non-saturable accumulation as diffusion, reasoning that lipid bilayers are (and need to be) essentially impervious to all molecules [46]. Kell reasons that alien molecules (such as drugs or agrochemicals) enter cells as low affinity orphan substrates of other protein transporters and channels, mainly because they resemble natural metabolites. It seems likely that orphan carriers account for most or all of the diffusion terms in uptake models. This is good news for auxins as herbicides because we know that mutations in AUX1 can confer e.g., 2,4-D resistance [28]. Resistance to auxin herbicides is rising [47], but it is unlikely that this will ever arise as a result of mutations in auxin uptake carriers because even with a defective AUX1 these compounds will arrive inside plant cells.

## 7. Evolutionary Analysis of AUX1

**Figure 4:**
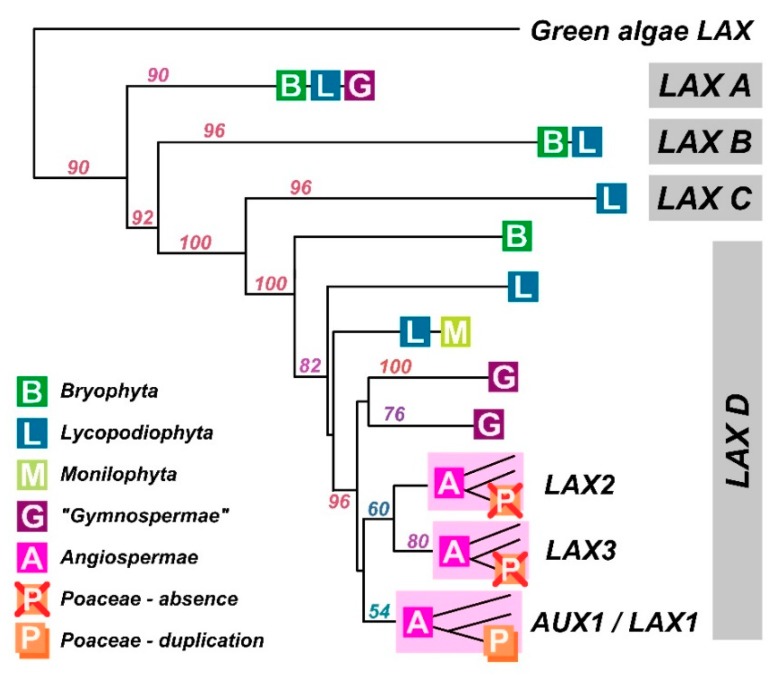
The AUX1/LAX phylogenetic tree. A summary of the maximum-likelihood phylogenetic analysis of nucleotide sequences using a GTR + G + I evolutionary model. Major taxonomic groups are depicted as letters. Node captions indicate bootstrap values.

Our understanding of PIN proteins has benefitted from a very thorough evaluation of their ancestry [48]. Bioinformatics on AUX1 [49] suggests that AUX1/LAX proteins arose before the evolution of land plants because AUX1/LAX-like sequences are present in Chlorophytes such as *Chlorella* and *Coccomyxa*. However, they are absent from most Chlorophyta and Streptophyta, and there is no functional evidence that these rather distant ancestral relatives are auxin transporters, although we can’t exclude the possibility. After analysing the alignments of many eudicot AUX1 protein sequences with *Chlorella variaialis,* we found only 18–28% sequence similarity (Figure 4; Figure S1). Amongst the monocots and eudicots, similarity is 70–86%. Nevertheless, it is important to note that despite low similarity, the green algal AUXs form a monophyletic cluster inside the tree of all amino acid permeases, making these algal AUXs distant, but true orthologues.

All land plant AUX1/LAX proteins are highly conserved (Figure 2 and Figure 4). Homologous sequences are consistent in length and lack variable loops, and the greatest variability can be found in N- and C-terminal parts (Figure 2). The phylogenetic tree shows that the family underwent a massive radiation in the early stages of land plant evolution. An ancestral clade (which we refer to as LAX A) is conserved across land plants before being lost in angiosperms. Two clades, (LAX B and LAX C) are found only in mosses and lycopods and, for example, LAX C consists of 12 members only in *Selaginella moellendorffii*. As discussed about the algae above, there is no functional evidence that auxin is the substrate for the orthologues in these ancestral clades, although they clearly accumulate features which persist in AUX1 (the group referred to as LAX D). All homologues in *Arabidopsis thaliana* (AUX1, LAX1, LAX2 and LAX3) belong to clade LAX D which underwent to at least two independent radiations, firstly, to a duplication in gymnosperms and secondly, to a triplication in early angiosperms resulting in tree clades, AUX1/LAX1, LAX2 and LAX3. All these three clades are represented in the genome of the basal angiosperm *Amborella trichopoda*. Points of note from phylogenetic analysis include the distinctive clade of AUX1-like proteins in the gymnosperms and the loss of both LAX2 and LAX3 clades in the Poaceae, although the Poaceae had an additional duplication event in the AUX1 clade (and homologues of LAX2 and LAX3 are present in other monocots like banana, asparagus and garlic). 

## 8. Sequence and Structure

We have learnt a lot about the biochemistry of AUX1, but little is known about its structure. As soon as the AUX1 sequence was known it was challenged with algorithms to predict secondary structure. These suggested 10, 11 or 12 transmembrane domains linked by variable length intra- and extracellular loops [30]. The AUX1/LAXs are part of the amino acid permease superfamily which, characteristically, have 12 transmembrane domains [50], but topological features suggested that 11 was the most likely number for AUX1 [30] and topology predictions continue to model AUX1 with 11 transmembrane domains (Figure 2). The variable N- and C-termini are positioned in the cytoplasm and apoplast, respectively. The sites of known mutations lie within both helical transmembrane domains and in the loops between membrane helices. 

Unfortunately, sequence identity to a protein for which a structure has been solved is so low that homology modelling is unrealistic at present (16% identity to a zebrafish sodium/neutral amino acid transporter). Nevertheless, the topology predictions allow some comparison to the accumulating knowledge of mammalian co-transporter proteins [51]. There are indications of an inverted two-fold symmetry of the transmembrane helices with 5 + 6 helical bundles. This follows the pattern of other symporters which use an inwardly directed proton electrochemical gradient to drive uptake. The membrane-spanning helices are likely to drive an alternating-access mechanism [52]. Thus, when IAA and protons bind to an outward-open conformation there is a change in helix arrangement giving rise to an inward-open conformation from which the bound substrates dissociate as the protons dissipate in the proton-poor cytoplasm. However, such predictions require crystallography results for validation.

Very few residues are conserved between the putative Chlorophyta orthologues and land plant sequences (Figure 2b), but there is a high degree of conservation (identity) once AUX1 and the LAX proteins evolved. Most of the conservation is in the transmembrane helices, although there are also islands of high conservation in loops 2–3, 3–4, 6–7 and 7–8 (Figure 2a). There are very tight turns between helices 1–2, 4–5 and 9–10 which may indicate contributions to the inverted cone morphologies assumed by stacked transmembrane helices seen with other co-transporters [51]. It is the small movements of these helical modules that drive transport across the membrane, although this kinetic model remains unproven for AUX1.

## 9. AUX1 kinetics and Models of Auxin Action

The speed of auxin movement has been measured over many years (summarised in [53]) and the polar transport element is dominated by the activity of efflux transporters, particularly PINs [1,54]. Nevertheless, many developmental auxin gradients are dependent on AUX1, such as the patterns of auxin distribution in the *Arabidopsis* root apex and during gravitropism [55,56]. These auxin distributions have been modelled using a variety of mathematical approaches. Some models do not include AUX1 (e.g., [57]), some allow sufficient influx without further consideration of AUX1 (e.g., [58]) and some develop proxy values of auxin influx as part of parameterisation (e.g., [55]). In a few models, the contribution of uptake via AUX1 (uptake permeability, or P_AUX_) is specified even though assumed large, apolar and invariant (e.g., [59]; P_AUX_ = 30 μm/s; [58]; P_AUX_ = 20 μm/s), or it is specified and much lower from a value inferred from measurements by Delbarre et al., [38] with BY-2 tobacco tissue cultured cells [56] (P_AUX1_ = 0.2 cm/h, 0.56 μm/s).

Despite the variability of approaches used for modelling, kinetic values for auxin influx through AUX1 have been measured. The accumulation of radiolabelled 2,4-D into tobacco BY-2 cells gave a calculated “influx carrier transport activity” of 1.4 μm/s [40]. Similar uptake measurements of radiolabelled IAA into *Arabidopsis* protoplasts gave a value for uptake permeability of 1.5 μm/s [60]. In both these careful studies, AUX1 activity was shown to dominate auxin influx, showing that the contribution of ill-defined diffusive permeability is small *in planta*. Further, the contribution of diffusion to the total flux of IAA was relevant to influx only during a loading phase (a rise in extracellular auxin concentration), and that it was insignificant for influx once the cells were at equilibrium [40]. Interestingly, the permeability values for protoplasts [60] and cells with cell walls [40] are similar and it is reassuring that two independent evaluations of influx permeability arrive at very similar values (1.4 and 1.5 μm/s). The dominance of AUX1 activity for auxin influx also explains how the assumption of a high and invariant influx permeability had no penalty in the outcomes of earlier models, and the uniformity of the measured permeability values from different cell types also suggests that the single uniform parameter entered into models for whole organs and whole plants is realistic. 

All system models noted above focused on idealised primary root growth. However, as noted above, there are phenotypes that are dependent on a wild type AUX1 activity, including root gravitropism. Clearly, a high permissive uptake permeability value will not explain phenotypes dependent on AUX1. Several of the models do estimate carrier abundance from micrographs and add these to cell maps (e.g., [56]) and these clearly add value to help fit simulations to phenotypes. Additionally, the more recent and comprehensive models of auxin distribution do parameterise AUX1 uptake activity with more care and these are adding value to our understanding of familiar phenotypes. For example, the model of Band and colleagues has helped to indicate that auxin asymmetry after gravistimulation is engaged very rapidly, within minutes, illustrating the power of the auxin transport machinery. Additionally, the spatiotemporal model of Moore et al., [61] uses the uptake permeability value from Rutschow et al., [60] and illustrates that AUX1 activity is integral to auxin patterning and, importantly, both PIN and AUX1 activities need to be coordinated for correct root development [61]. Accurate kinetic understanding of AUX1 is vital for valid system models.

## 10. Summary and Gaps in Our Knowledge

It has taken over 40 years to add a significant new understanding to the description of auxin uptake provided by Rubery and Sheldrake [17]. AUX1 may not have the dynamic cell biology of the PIN proteins, but it is vital to the formation of auxin gradients and, hence, to patterning in plants. Indeed, the accumulation of IAA inside cells is necessary for PIN-mediated redistribution because, if PIN proteins are channels they can only deliver auxin down an auxin concentration gradient. We now have accurate and reliable figures for the affinity of AUX1 for IAA (0.5 μM; [44]) and for AUX1’s permeability (1.4 μm/s; [40]) and these can be used with confidence by systems modellers. We also have 3-dimensional pharmacophoric maps for tobacco AUX1 [39]. It is clear that the ancestry of AUX1 and the LAXs arises with the land plants (Figure 2), although there may have been some chlorophytes with uptake carriers for organic acids, including IAA [49]. 

What remains unclear is how the LAX2/3s differ from the AUX1/LAX1 clade, although tissue specific expression may be sufficient explanation for this diversification of the family. We also lack functional detail on how the clades differ in functionality. The loss of LAX2/3s in the grasses is intriguing, given that the basis of selectivity of auxins as broad-leaved herbicides remains to be elucidated. However, the biggest outstanding questions are around the mechanism of action of these transporters, and these questions will only be resolved by solving the structure of AUX1 (or one of the LAXes) in both loading and unloading orientations. Only then will we start to understand the basis of how these transporters select for IAA over the many other organic acids available in plant apoplastic fluids, and how, once selected, this molecule is moved across the plasma membrane against a concentration gradient powered by two protons.

## Supplementary Materials

Supplementary materials can be found at http://www.mdpi.com/1422-0067/19/11/3391/s1.

## References

[1] Adamowski M., Friml J. (2015). PIN-dependent auxin transport: Action, regulation, and evolution. Plant Cell.

[2] Lacek J., Retzer K., Luschnig C., Zažímalová E. (2017). Polar Auxin Transport. ELS.

[3] Swarup R., Péret B. (2012). AUX/LAX family of auxin influx carriers-an overview. Front Plant Sci..

[4] Terasaka K., Blakeslee J.J., Titapiwatanakun B., Peer W.A., Bandyopadhyay A., Makam S.N., Lee O.R., Richards E.L., Murphy A.S., Sato F. (2005). PGP4, an ATP binding cassette P-glycoprotein, catalyzes auxin transport in *Arabidopsis thaliana* roots. Plant Cell.

[5] Krouk G., Lacombe B., Bielach A., Perrine-Walker F., Malinska K., Mounier E., Hoyerova K., Tillard P., Leon S., jung K. (2010). Nitrate-regulated auxin transport by NRT1.1 defines a mechanism for nutrient sensing in plants. Dev. Cell.

[6] Kamimoto Y., Terasaka K., Hamamoto M., Takanashi K., Fukuda S., Shitan N., Sugiyama A., Suzuki H., Shibata D., Wang B. (2012). Arabidopsis ABCB21 is a facultative auxin importer/exporter regulated by cytoplasmic auxin concentration. Plant Cell Physiol..

[7] Kubeš M., Yang H., Richter G.L., Cheng Y., Młodzińska E., Wang X., Blakeslee J.J., Carraro N., Petrášek J., Zažímalová E. (2012). The Arabidopsis concentration-dependent influx/efflux transporter ABCB4 regulates cellular auxin levels in the root epidermis. Plant J..

[8] Swarup R., Friml J., Marchant A., Ljung K., Sandberg G., Palme K., Bennett M. (2001). Localization of the auxin permease AUX1 suggests two functionally distinct hormone transport pathways operate in the Arabidopsis root apex. Genes Dev..

[9] Marchant A., Kargul J., May S.T., Muller P., Delbarre A., Perrot-Rechenmann C., Bennett M.J. (1999). AUX1 regulates root gravitropism in Arabidopsis by facilitating auxin uptake within root apical tissues. EMBO J..

[10] Vandenbussche F., Petrásek J., Zádníková P., Hoyerová K., Pesek B., Raz V., Bennett M., Zažímalová E., Benková E., Van Der Straeten D. (2010). The auxin influx carriers AUX1 and LAX3 are involved in auxin-ethylene interactions during apical hook development in Arabidopsis thaliana seedlings. Development.

[11] Swarup K., Benková E., Swarup R., Casimiro I., Péret B., Yang Y., Parry G., Nielsen E., De Smet I., Vanneste S. (2008). The auxin influx carrier LAX3 promotes lateral root emergence. Nat. Cell Biol..

[12] Kasprzewska A., Carter R., Swarup R., Bennett M., Monk N., Hobbs J.K., Fleming A. (2015). Auxin influx importers modulate serration along the leaf margin. Plant J..

[13] Bhosale R., Giri J., Pandey B.K., Giehl R.F.H., Hartmann A., Traini R., Truskina J., Leftley N., Hanlon M., Swarup K. (2018). A mechanistic framework for auxin dependent Arabidopsis root hair elongation to low external phosphate. Nat Commun..

[14] Dindas J., Scherzer S., Roelfsema M.R.G., von Meyer K., Müller H.M., Al-Rasheid K.A.S., Palme K., Dietrich P., Becker D., Bennett M.J. (2018). AUX1-mediated root hair auxin influx governs SCF. Nat. Commun..

[15] Fendrych M., Akhmanova M., Merrin J., Glanc M., Hagihara S., Takahashi K., Uchida N., Torii K.U., Friml J. (2018). Rapid and reversible root growth inhibition by TIR1 auxin signalling. Nat. Plants.

[16] O’Leary B.M., Neale H.C., Geilfus C.M., Jackson R.W., Arnold D.L., Preston G.M. (2016). Early changes in apoplast composition associated with defence and disease in interactions between *Phaseolus vulgaris* and the halo blight pathogen *Pseudomonas syringae* Pv. phaseolicola. Plant Cell Environ..

[17] Rubery P.H., Sheldrake A.R. (1974). Carrier-mediated auxin transport. Planta.

[18] Parry G., Delbarre A., Marchant A., Swarup R., Napier R., Perrot-Rechenmann C., Bennett M.J. (2001). Novel auxin transport inhibitors phenocopy the auxin influx carrier mutation aux1. Plant J..

[19] Rubery P.H., Sheldrake A.R. (1973). Effect of pH and surface charge on cell uptake of auxin. Nat. New Biol..

[20] Jacobs M., Hertel R. (1978). Auxin binding to subcellular fractions from Cucurbita hypocotyls: In vitro evidence for an auxin transport carrier. Planta.

[21] Hertel R., Lomax T.L., Briggs W.R. (1983). Auxin transport in membrane vesicles from *Cucurbita pepo* L.. Planta.

[22] Lomax T.L., Mehlhorn R.J., Briggs W.R. (1985). Active auxin uptake by zucchini membrane vesicles: Quantitation using ESR volume and delta pH determinations. Proc. Natl. Acad. Sci. USA.

[23] Benning C. (1986). Evidence supporting a model of voltage-dependent uptake of auxin into Cucurbita vesicles. Planta.

[24] Geier U., Werner O., Bopp M. (1990). Indole-3-acetic acid uptake in isolated protoplasts of the moss *Funaria hygrometrica*. Planta.

[25] Barbez E., Dünser K., Gaidora A., Lendl T., Busch W. (2017). Auxin steers root cell expansion via apoplastic pH regulation in. Proc. Natl. Acad. Sci. USA.

[26] Inoue S.I., Takahashi K., Okumura-Noda H., Kinoshita T. (2016). Auxin Influx Carrier AUX1 Confers Acid Resistance for Arabidopsis Root Elongation Through the Regulation of Plasma Membrane, H+-ATPase. Plant Cell Physiol..

[27] Bennett M.J., Marchant A., Green H.G., May S.T., Ward S.P., Millner P.A., Walker A.R., Schulz B., Feldmann K.A. (1996). Arabidopsis *AUX1* gene: A permease-like regulator of root gravitropism. Science.

[28] Maher E.P., Martindale S.J. (1980). Mutants of Arabidopsis thaliana with altered responses to auxins and gravity. Biochem. Genet..

[29] Okada K., Shimura Y. (1990). Reversible root tip rotation in Arabidopsis seedlings induced by obstacle-touching stimulus. Science.

[30] Swarup R., Kargul J., Marchant A., Zadik D., Rahman A., Mills R., Yemm A., May S., Williams L., Millner P. (2004). Structure-function analysis of the presumptive Arabidopsis auxin permease AUX1. Plant Cell.

[31] Péret B., Swarup K., Ferguson A., Seth M., Yang Y., Dhondt S., James N., Casimiro I., Perry P., Syed A. (2012). AUX/LAX genes encode a family of auxin influx transporters that perform distinct functions during Arabidopsis development. Plant Cell.

[32] Swarup R., Kramer E.M., Perry P., Knox K., Leyser H.M., Haseloff J., Beemster G.T.S., Bhalerao R., Bennett M.J. (2005). Root gravitropism requires lateral root cap and epidermal cells for transport and response to a mobile auxin signal. Nat. Cell Biol..

[33] Kramer E.M., Bennett M.J. (2006). Auxin transport: A field in flux. Trends Plant Sci..

[34] Omasits U., Ahrens C.H., Müller S., Wollscheid B. (2014). Protter: Interactive protein feature visualization and integration with experimental proteomic data. Bioinformatics.

[35] Yang Y., Hammes U.Z., Taylor C.G., Schachtman D.P., Nielsen E. (2006). High-affinity auxin transport by the AUX1 influx carrier protein. Curr. Biol..

[36] Carrier D.J., Bakar N.T., Swarup R., Callaghan R., Napier R.M., Bennett M.J., Kerr I.D. (2008). The binding of auxin to the Arabidopsis auxin influx transporter AUX1. Plant Physiol..

[37] Lanková M., Smith R.S., Pesek B., Kubes M., Zazímalová E., Petrásek J., Hoyerová K. (2010). Auxin influx inhibitors 1-NOA, 2-NOA, and CHPAA interfere with membrane dynamics in tobacco cells. J. Exp. Bot..

[38] Delbarre A., Muller P., Imhoff V., Guern J. (1996). Comparison of mechanisms controlling uptake and accumulation of 2,4-dichlorophenoxy acetic acid, naphthalene-1-acetic acid, and indole-3-acetic acid in suspension-cultured tobacco cells. Planta.

[39] Imhoff V., Muller P., Guern J., Delbarre A. (2000). Inhibitors of the carrier-mediated influx of auxin in suspension-cultured tobacco cells. Planta.

[40] Hošek P., Kubes M., Lanková M., Dobrev P.I., Klíma P., Kohoutová M., Petrášek J., Hoyerová K., Jiřina M., Zažímalová E. (2012). Auxin transport at cellular level: New insights supported by mathematical modelling. J. Exp. Bot..

[41] Simon S., Kubeš M., Baster P., Robert S., Dobrev P.I., Friml J., Petrášek J., Zažímalová E. (2013). Defining the selectivity of processes along the auxin response chain: A study using auxin analogues. New Phytol..

[42] Sussman M.R., Goldsmith M.H. (1981). Auxin uptake and action of N-1-naphthylphthalamic acid in corn coleoptiles. Planta.

[43] Tsuda E., Yang H., Nishimura T., Uehara Y., Sakai T., Furutani M., Koshiba T., Hirose M., Nozaki H., Murphy A.S. (2011). Alkoxy-auxins are selective inhibitors of auxin transport mediated by PIN, ABCB, and AUX1 transporters. J. Biol. Chem..

[44] Hoyerova K., Hosek P., Quareshy M., Li J., Klima P., Kubes M., Yemm A.A., Neve P., Tripathi A., Bennett M.J. (2018). Auxin molecular field maps define AUX1 selectivity: Many auxin herbicides are not substrates. New Phytol..

[45] Quareshy M., Prusinska J., Kieffer M., Fukui K., Pardal A.J., Lehmann S., Schafer P., del Genio C.I., Kepinski S., Hayashi K. (2018). The Tetrazole Analogue of the Auxin Indole-3-acetic Acid Binds Preferentially to TIR1 and Not AFB5. ACS Chem. Biol..

[46] Kell D.B. (2016). Implications of endogenous roles of transporters for drug discovery: Hitchhiking and metabolite-likeness. Nat. Rev. Drug Discov..

[47] Busi R., Goggin D.E., Heap I., Horak M.J., Jugulam M., Masters R.A., Napier R.M., Riar D.S., Satchivi N.M., Torra J. (2018). Weed resistance to synthetic auxin herbicides. Pest Manag. Sci..

[48] Bennett T., Brockington S.F., Rothfels C., Graham S.W., Stevenson D., Kutchan T., Rolf M., Thomas P., Wong G.K.-S., Leyser O. (2014). Paralogous radiations of PIN proteins with multiple origins of noncanonical PIN structure. Mol. Biol. Evol..

[49] De Smet I., Voss U., Lau S., Wilson M., Shao N., Timme R.E., Swarup R., Kerr I., Hodgman C., Bock R. (2011). Unraveling the evolution of auxin signaling. Plant Physiol..

[50] Fischer W.N., Kwart M., Hummel S., Frommer W.B. (1995). Substrate specificity and expression profile of amino acid transporters (AAPs) in Arabidopsis. J. Biol. Chem..

[51] Fowler P.W., Orwick-Rydmark M., Radestock S., Solcan N., Dijkman P.M., Lyons J.A., Kwok J., Caffrey M., Watts A., Forrest L.R. (2015). Gating topology of the proton-coupled oligopeptide symporters. Structure.

[52] Jardetzky O. (1996). Protein dynamics and conformational transitions in allosteric proteins. Prog. Biophys. Mol. Biol..

[53] Kramer E.M., Rutschow H.L., Mabie S.S. (2011). AuxV: A database of auxin transport velocities. Trends Plant Sci..

[54] Naramoto S. (2017). Polar transport in plants mediated by membrane transporters: Focus on mechanisms of polar auxin transport. Curr. Opin. Plant Biol..

[55] Band L.R., Wells D.M., Fozard J.A., Ghetiu T., French A.P., Pound M.P., Wilson M.H., Yu L., Li W., Hijazi H.I. (2014). Systems analysis of auxin transport in the Arabidopsis root apex. Plant Cell.

[56] Band L.R., Wells D.M., Larrieu A., Sun J., Middleton A.M., French A.P., Brunoud G., Sato E.M., Wilson M.H., Péret B. (2012). Root gravitropism is regulated by a transient lateral auxin gradient controlled by a tipping-point mechanism. Proc. Natl. Acad. Sci. USA.

[57] Mironova V.V., Omelyanchuk N.A., Novoselova E.S., Doroshkov A.V., Kazantsev F.V., Kochetov A.V., Kolchanov N.A., Mjolsness E., Likhoshvai V.A. (2012). Combined in silico/in vivo analysis of mechanisms providing for root apical meristem self-organization and maintenance. Ann. Bot..

[58] Grieneisen V.A., Xu J., Marée A.F., Hogeweg P., Scheres B. (2007). Auxin transport is sufficient to generate a maximum and gradient guiding root growth. Nature.

[59] Wabnik K., Kleine-Vehn J., Balla J., Sauer M., Naramoto S., Reinöhl V., Merks R.M., Govaerts W., Friml J. (2010). Emergence of tissue polarization from synergy of intracellular and extracellular auxin signaling. Mol. Syst. Biol..

[60] Rutschow H.L., Baskin T.I., Kramer E.M. (2014). The carrier AUXIN RESISTANT (AUX1) dominates auxin flux into Arabidopsis protoplasts. New Phytol..

[61] Moore S., Liu J., Zhang X., Lindsey K. (2017). A recovery principle provides insight into auxin pattern control in the Arabidopsis root. Sci. Rep..

