# Supplementary material for: Advances in Understanding the Mechanism of Action of the Auxin Permease AUX1
Source: Int J Mol Sci. 2018 Oct 30;19(11):3391. doi: 10.3390/ijms19113391 (PMC6275028; doi:10.3390/ijms19113391)
Supplement: Supplementary file 1 [file ijms-19-03391-s001.zip › Singh et al Suppl/SinghetalSuppl.docx]

**Supporting information:**

Phylogeny of the AUX1/LAX family: Methods.

Protein and corresponding coding sequences of AUX1/LAX homologues were obtained by BLAST searches on Phytozome [1] using blastp [2] against the protein database using AtAUX1 as a query. Peptide sequences were aligned by MAFFT using default settings [3] and the resulting alignment was used as a template for nucleotide alignment of coding sequences constructed using the pal2nal utility [4]. Additional transcriptomic sequences were obtained by tblastn searches on the 1KP server [5] and hits were added to the existing alignment by the MAFFT --add procedure. Only full-length sequences were used for phylogenetic analysis. Variable N- and C-terminal regions and gapped sites with coverage lower than 80 % were excluded from the analysis. Phylogenetic analysis was performed by MEGAX software [6] using Maximum Likelihood method based on General Time Reversible evolutionary model [7] with discrete Gamma distribution of evolutionary rates and invariable sites (GTR+G+I). The phylogenetic tree was validated by 500 bootstrap replications.

**SI Figure1.** Maximum-likelihood phylogenetic analysis of nucleotide sequences of AUX1/LAX family members using a GTR+G+I evolutionary model. Node captions indicate bootstrap values.

**References**

1. Goodstein DM, Shu S, Howson R, Neupane R, Hayes RD, Fazo J, et al. Phytozome: a comparative platform for green plant genomics. Nucleic Acids Res. 2012;40(Database issue):D1178-86. Epub 2011/11/22. doi: 10.1093/nar/gkr944.
2. Altschul SF, Gish W, Miller W, Myers EW, Lipman DJ. Basic local alignment search tool. J Mol Biol. 1990;215(3):403-10. doi: 10.1016/S0022-2836(05)80360-2.
3. Katoh K, Rozewicki J, Yamada KD. MAFFT online service: multiple sequence alignment, interactive sequence choice and visualization. Brief Bioinform. 2017. Epub 2017/09/06. doi: 10.1093/bib/bbx108.
4. Suyama M, Torrents D, Bork P. (2006) PAL2NAL: robust conversion of protein sequence alignments into the corresponding codon alignments. Nucleic Acids Res. 34, W609-W612.Wickett NJ, Mirarab S, Nguyen N, Warnow T, Carpenter E, Matasci N, et al. Phylotranscriptomic analysis of the origin and early diversification of land plants. Proc Natl Acad Sci U S A. 2014;111(45):E4859-68. Epub 2014/10/29. doi: 10.1073/pnas.1323926111.
5. Wickett NJ, ... Wong GKS, Leebens-Mack J. (2014) Phylotranscriptomic analysis of the origin and early diversification of land plants. Proc. Natl. Acad. Sci. USA 111: E4859-E4868.
6. Kumar S, Stecher G, Li M, Knyaz C, Tamura K. MEGA X: Molecular Evolutionary Genetics Analysis across Computing Platforms. Mol Biol Evol. 2018;35(6):1547-9. doi: 10.1093/molbev/msy096.
7. Nei M, Kumar S. (2000) Molecular Evolution and Phylogenetics. Oxford University Press, New York.
