# Supplementary figures and images for: Advances in Understanding the Mechanism of Action of the Auxin Permease AUX1
Source: Int J Mol Sci. 2018 Oct 30;19(11):3391. doi: 10.3390/ijms19113391 (PMC6275028; doi:10.3390/ijms19113391)

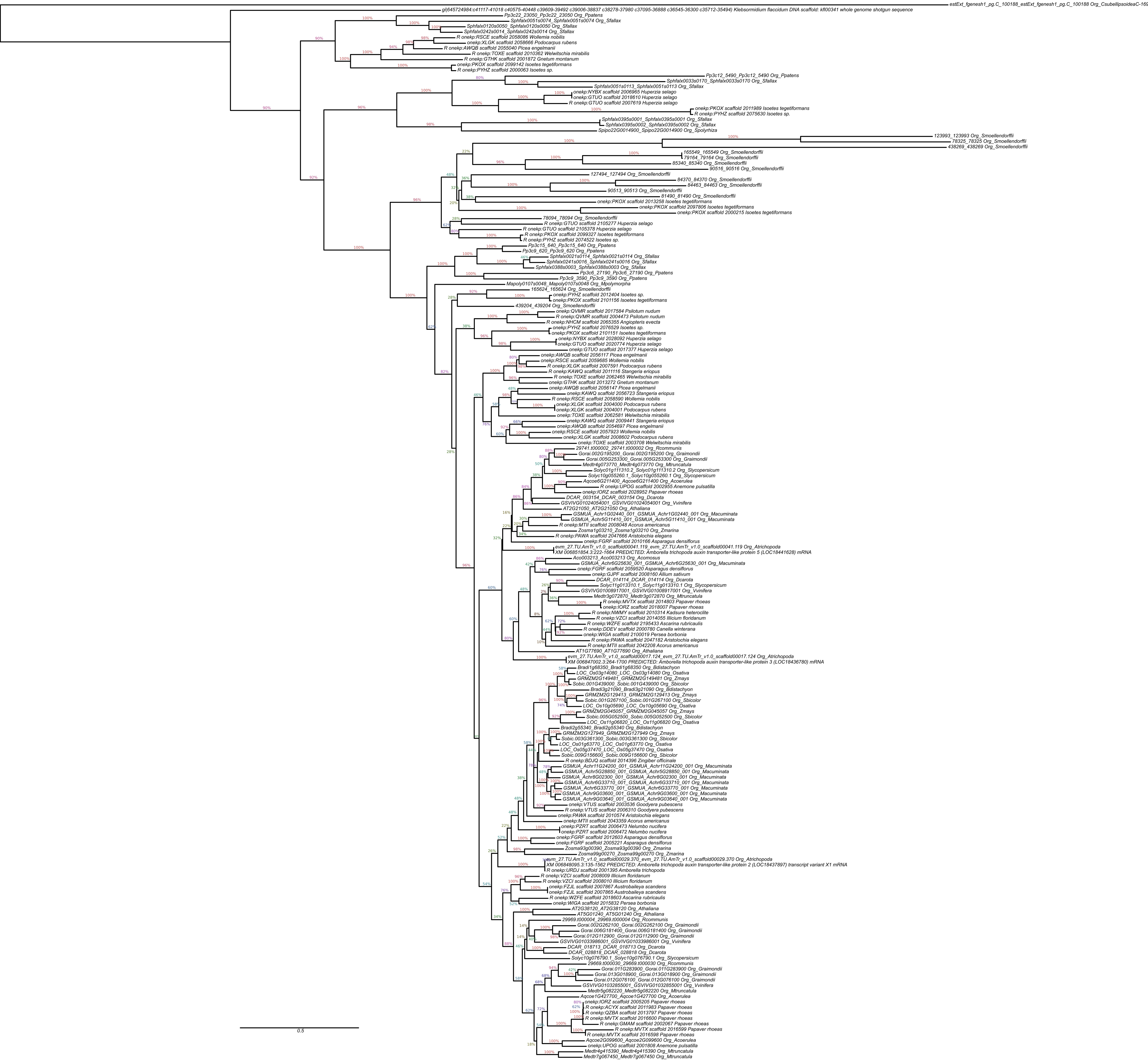

Supplement: Supplementary file 1 [file ijms-19-03391-s001.zip › Singh et al Suppl/SupplFig1.pdf]
